# The Effect of the Particle Size Reduction on the Biorelevant Solubility and Dissolution of Poorly Soluble Drugs with Different Acid-Base Character

**DOI:** 10.3390/pharmaceutics15010278

**Published:** 2023-01-13

**Authors:** Dóra Csicsák, Rita Szolláth, Szabina Kádár, Rita Ambrus, Csilla Bartos, Emese Balogh, István Antal, István Köteles, Petra Tőzsér, Vivien Bárdos, Péter Horváth, Enikő Borbás, Krisztina Takács-Novák, Bálint Sinkó, Gergely Völgyi

**Affiliations:** 1 Department of Pharmaceutical Chemistry, Semmelweis University, 9 Hőgyes Endre Street, 1092 Budapest, Hungary; 2 Department of Organic Chemistry and Technology, Faculty of Chemical Technology and Biotechnology, Budapest University of Technology and Economics, 3 Műegyetem Rkp., 1111 Budapest, Hungary; 3 Institute of Pharmaceutical Technology and Regulatory Affairs, University of Szeged, 6 Eötvös Street, 6720 Szeged, Hungary; 4 Department of Pharmaceutics, Semmelweis University, 7 Hőgyes Endre Street, 1092 Budapest, Hungary; 5 Pion Inc., 10 Cook Street, Billerica, MA 01821, USA

**Keywords:** particle size, solubility, dissolution, real-time monitoring, biorelevant media

## Abstract

Particle size reduction is a commonly used process to improve the solubility and the dissolution of drug formulations. The solubility of a drug in the gastrointestinal tract is a crucial parameter, because it can greatly influence the bioavailability. This work provides a comprehensive investigation of the effect of the particle size, pH, biorelevant media and polymers (PVA and PVPK-25) on the solubility and dissolution of drug formulations using three model compounds with different acid-base characteristics (papaverine hydrochloride, furosemide and niflumic acid). It was demonstrated that micronization does not change the equilibrium solubility of a drug, but it results in a faster dissolution. In contrast, nanonization can improve the equilibrium solubility of a drug, but the selection of the appropriate excipient used for nanonization is essential, because out of the two used polymers, only the PVPK-25 had an increasing effect on the solubility. This phenomenon can be explained by the molecular structure of the excipients. Based on laser diffraction measurements, PVPK-25 could also inhibit the aggregation of the particles more effectively than PVA, but none of the polymers could hold the nanonized samples in the submicron range until the end of the measurements.

## 1. Introduction

In recent years, the number of molecules belonging to BCS class II (low solubility, high permeability) and class IV (low solubility, low permeability) increased significantly, therefore improving the solubility of poorly soluble drugs became one of the most important challenges of the pharmaceutical industry [1,2]. Nowadays, several different solubility-enhancing techniques are available. There are methods based on physical changes such as particle size reduction, change of crystallinity (polymorphous or amorphous) or solid dispersion formation. Both physical and also chemical methods such as pH modification, complex or salt formation, or mixed methods such as supercritical fluid preparation or use of different solubilizing agents are known [3,4].

Particle-size reduction is one of the most commonly used processes in the formulation development. Due to the size reduction, the surface area of the particles increases, allowing a greater interaction with the solvent and improving the dissolution. There are two major types of size reduction based on the dimension of the size of the particle: micronization (particles between 1–1000 µm) and nanonization (particles in the submicron range, <1 µm) [5]. Micronization is a widely used method to enhance the bioavailability of an API. It is usually performed by milling (jet/ball milling) or high-pressure homogenization. Equilibrium solubility is not affected by micronization, but the dissolution rate will improve [3,6]. In contrast, nanonization can affect both the solubility and the dissolution rate, because under the critical 1µm size limit, the equilibrium solubility of a compound is not independent from the surface area anymore [7]. This can explain, why not only the velocity of the dissolution, but also the concentration of the saturated solution increases [8]. There are two major types of methods to approach nano-sized particles: top-down and bottom-up techniques. Top-down techniques such as milling or homogenization are producing very small particles, but they require stabilizers (most commonly polymers), and the heat forming during the process can cause damage in the substance. Precipitation or sol-gel formation belongs to the bottom-up techniques. These methods are mostly cost efficient and they result in smaller particle size with a narrow size distribution. However, the substance should be soluble at least in one solvent, and the product can contain solvent residues [5,8,9,10].

Knowing the accurate solubility of a drug in the early stages of development is essential for a successful product. There are many factors that can influence this parameter, including but not limited to: pH and degree of ionization, solubilizing agents, temperature, particle size and crystalline structure [11,12,13]. To obtain such solubility data, which is important in terms of bioavailability, buffers with a pH that is relevant in the gastrointestinal tract should be applied. Usually, pH = 1.2–1.6 buffers are used to simulate the gastric media, pH = 6.5 buffers are commonly used to imitate the fasted-state intestinal pH conditions, while pH = 5.0 buffers help to mimic fed-state intestinal conditions [14,15]. However, the adjusted pH alone does not describe the actual situation in the gastrointestinal tract, therefore using biorelevant media (BRM) can provide a more precise outcome. These media were invented to model additional important properties of the gastrointestinal fluids, including solubilization by bile salts and lecithin. These agents can solubilize the molecules resulting in a higher solubility [15,16]. The equilibrium solubility of a compound can be measured by several different techniques, however the “gold standard” is still the saturation shake-flask method (SSF) [17]. After reaching the equilibrium, where the solid and the solvent phases are in balance, the concentration of the saturated solution provides the equilibrium solubility, but the remaining solid phase also should be analyzed to reveal the possible polymorph or salt transformations [18,19].

In our previous work, we determined the biorelevant solubility of four drugs representing different acid-base characters and studied the effect of pH, solubilizing agents and the food effect for the compounds, rivaroxaban, furosemide, papaverine and niflumic acid. In that study, the solubility was measured by the SSF method, but the solid phase was not analyzed [20].

In this work, we provide a more comprehensive investigation of factors, which can influence not only the solubility but the dissolution in BRM. The first goal of this study was to prepare size-reduced samples with a simple and commonly used technique, dry milling. Furthermore, the aim of this work was to investigate the effect of the particle size and the effect of the different characteristics of the excipients on solubility and dissolution. In addition, the solid particles were analyzed from the preparation until the end of the solubility measurements using different analytical methods. Finally, the relation between the solubility results and the molecular characteristic of the polymer excipients was confirmed.

The model compounds are furosemide (BCS IV) as an acid, papaverine hydrochloride (BCS II) as a salt of a base and niflumic acid (BCS II) as an ampholyte [20]. From the commercially available substances (named here as original or starting materials) micro- and nanonized forms were prepared. In the preparation of nano-sized products, polymer excipients were used to prevent the aggregation of the particles. The solubility of all samples was measured in a phosphate buffer with pH = 6.5 (FaSSIF blank) and an acetate buffer with pH = 5.0 (FeSSIF blank) and in the biorelevant dissolution media (FaSSIF and FeSSIF full) (biorelevant). In the case of the original substances and the micronized forms, all the solubility and dissolution measurements were performed with the pure substance and in the presence of the excipients used for nanonization, therefore the possible effect of these excipients was also studied. The solid-state analysis at the end of the solubility measurements was performed with powder X-Ray Diffraction (PXRD). After the solubility measurements, the particle size of the solid phase from the sample was measured with a laser diffraction particle size analyzer (Mastersizer^TM^).

## 2. Materials and Methods

### 2.1. Materials

Niflumic acid (NIF) was purchased from Sigma-Aldrich Co. Llc. (St. Louis, MO, USA), papaverine hydrochloride (PAP) from Molar Chemicals Ltd. (Halásztelek, Hungary), and furosemide (FUR) from TCI Europe N.V. (Haven, Belgium). From the commercially available substances micro- and nano-sized products were prepared by milling (Retsch Ball Mill (Retsch Hungary, Budapest, Hungary), 400 rpm, 2 h) at the University of Szeged. The milling time and rpm were identical for micronization and nanonization, but for nano-sized products the polymer excipients were added in 1 to 1 mass ratio. Two different polymers were used: polyvinyl alcohol (PVA) and polyvinylpyrrolidone-25 (PVPK-25) purchased from Sigma-Aldrich Co. Llc. (St. Louis, MO, USA). The distilled water of Ph. Eur. grade was used. All other reagents (sodium chloride, sodium hydroxide pellets, acetic acid, sodium dihydrogen phosphate) were of analytical grade. SIF powder was purchased from Biorelevant (London, UK).

### 2.2. Solubility Measurements

The equilibrium solubility of the samples was determined by the SSF method. The concentrations for the blank buffers were available at the manufacturer. FaSSIF full and FeSSIF full buffers were prepared from instant SIF powder added to the blank buffers and could be used for 48 h. For FaSSIF, a full 0.224 g of SIF powder was dissolved in 100 mL of blank phosphate buffer (pH = 6.5). After 2 h, a slightly opalesque solution was formed and it meant that it was ready for the measurement. For FeSSIF, a full 1.12 g of SIF powder was dissolved in 100 mL of blank acetate buffer (pH = 5.0) and was ready to use. The samples were added in excess to the aqueous buffer solutions to produce a suspension. To measure the original samples and the micronized forms in the presence of the excipients, the same 1 to 1 mass ratio was used as in the case of nanonized samples: a physical mixture was prepared from the API and the PVA or PVPK-25. The composition of the investigated formulations and their nomination in the following can be found in Table 1.

The prepared suspensions were vigorously stirred for 6 h at a controlled 37.0 ± 0.1 °C temperature followed by a sedimentation period of 18 h. In special cases, where reaching the equilibrium lasted longer than 24 h, a longer measurement time was used. The concentration of the saturated solutions was determined by UV spectroscopy using a Jasco V-550 UV/VIS spectrophotometer. The aliquots taken out from solubility experiments were diluted if necessary with the buffer used for the given solubility measurement, and the absorbance was measured at the *λ_max_*. The same buffer was used as a blank solution during the spectrophotometric measurements. To calculate the concentration from the measured absorption values, the specific absorbance ($A_{1\mathrm{cm}}^{1\%}$, the absorbance of 1 g/100 mL solution over a 1 cm optical pathlength at a given wavelength) of the sample in each media was determined separately at the selected wavelength using a dilution series, from the linear regression equation (Lambert–Beer law). Representatively, as an example, the calibration curve of furosemide in FaSSIF blank buffer is shown on Figure S1 (Supplementary Materials). Three parallel measurements were performed for each API with or without excipients in all media.

### 2.3. In situ Dissolution Measurements

The dissolution measurements were performed using the same buffer solutions as in the case of solubility measurements. The concentration versus time relationship was investigated with an in situ procedure. The operation of the used device (µDISS Profiler^TM^, Pion Inc., Billerica, MA, USA) is also based on UV spectrophotometry, but through the connected fiber optic probes measuring the concentration in real-time is possible [21,22,23]. These fiber optic probes are inserted in 6 temperature-controlled (37.0 ± 0.1 °C) vessels, which were stirred with a magnetic stirrer. The tips of the probes influence the pathlength (2–5–10–20 mm), therefore they should be chosen according to the expected concentration. In cases where the concentration of the solution was out of the range of the device, the real-time monitoring of the dissolution was not possible. After adjusting the right tip to the probes, the UV spectra were registered according to the following protocol: 1 spectrum per 30 s in 0–4 h, 1 spectrum per 1 min in 4–12 h, and 1 spectrum per 2 min in 12–24 h. In cases where 24 h were not enough to reach the equilibrium, further data points were also registered. For the evaluation of the concentration, the calibration data and second derivative spectra were used. The calibration was performed in each media and for each UV probe separately.

### 2.4. Particle Size Analysis

The mean particle size and size distribution of the original compound and the reduced size substances were determined by Laser Diffractometry and Scanning Electron microscopy (SEM).

For Laser Diffractometry, 0.1 mL of milled suspensions were dispersed in 100 mL of demineralized water at a mixing speed of 1500 rpm with Mastersizer Hydro 2000 SM small volume dispersion unit. The particle size of the samples was measured at 25 ± 1 °C using a Mastersizer 2000 (Malvern Instruments Ltd., Malvern, UK). Laser diffraction measures the angular distribution of light scattered by the diluted sample and detects particles from 0.1 to 3000 μm. General purpose measurement with enhanced sensitivity mode was utilized, which was also useful for sample characterizations containing irregularly shaped particles. Every sample was measured three times individually, and the mean values were reported to track the changing of the particle size and span values (width of particle size distributions). Each measurement took 20 s to perform suggested by the Malvern diffraction application to allow slow-moving larger aggregates to pass through the detector array.

The particle size of the drug-nanoparticles—the particles were on the surface of the polymer microparticles—were investigated by SEM pictures (Hitachi S4700; Hitachi Ltd., Tokyo, Japan) at 10 kV. The distribution of the drug particle diameter was obtained by analyzing SEM images with the ImageJ software (1.50i; Java 1.6.0_20 [32-bit]; Windows NT) environment using approximately 500 particles.

### 2.5. PXRD

Powder X-Ray Diffraction patterns were used to verify the structure of the original compound, the micronized and nanonized product, and to examine the solid phase from the solubility measurements. To obtain these samples, a small amount of the solid phase at the end of the solubility measurement was isolated and dried on a glass plate. The measurement was carried out by a PANalytical (Amelo, The Netherlands) X’pert ProMDP X-ray diffractometer using Cu-Kα radiation (1.524 Ǻ) and a Ni filter. The applied voltage was 40 kV, while the current was 30 mA. The samples were analyzed between 4° and 42° 2Ɵ, except furosemide, because in that case, due to the place of the characteristic peaks, the samples should be investigated between 2° and 42° 2Ɵ.

## 3. Results and Discussion

Three compounds with different acid-base properties were chosen to study the particle-size reduction effect on BRM solubility and dissolution. The equilibrium solubility of the original substances, the micronized forms, their physical mixtures with excipients used for nanonization and two nanonized forms (altogether 24 samples) were measured by the standardized SSF method at 37 °C in four solvents (FaSSIF blank and full, FeSSIF blank and full). Their dissolution in the same media was measured by in situ real-time monitoring using the µDISS device. The particle size was analyzed by Laser Diffractometry and Scanning Electron microscopy. The particle size distribution of the original and the micronized compounds are shown in Table 2 using the d = 0.1, 0.5 and 0.9 values. These values show the upper limit of the size range in µm, under which 10%, 50% and 90% of the particles belong. We have to note that the original, commercially available substances were not macrocrystalline materials, their particles’ size were in the µm range, but size reduction by milling caused a significant decrease. These samples are referred to here as micronized.

SEM was used to investigate the structure and size range of the nanonized samples. The structures of the prepared substances are shown in Figure S2; their mean particle size is shown in Table S1. Based on these measurements, it is proved that all prepared nanonized samples are in the submicron range. The solid phase of the solubility experiments was analyzed by the PXRD method. The results are shown and discussed for each compound separately.

### 3.1. Papaverine Hydrochloride

#### 3.1.1. Solubility Measurements

**Figure 1:**
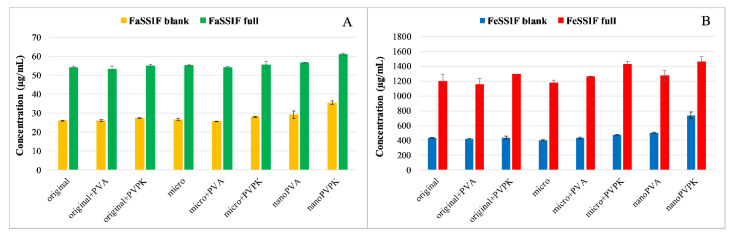
The solubility results of papaverine hydrochloride in pH = 6.5 (**A**) and pH = 5.0 (**B**) media.

The solubility results of papaverine hydrochloride are shown in Figure 1.

As a base with p*K*_a_ = 6.36, it is more soluble in pH = 5.0 media than in buffers of pH = 6.5, because in the first case 95.8% of the molecules are protonated, which results in an increased polarity and therefore an increased aqueous solubility [20,24]. The solubilizing effect of the SIF powder is prevailing in both the FaSSIF and FeSSIF full buffers, resulting in a higher solubility of the compound than in the blank buffers. Micronization, as expected, did not cause a significant difference in equilibrium solubility compared to the original substance. The nanonization has led to different results: nano-sized compounds with PVPK excipient (nanoPVPK) resulted in a higher solubility, but nanonization with PVA (nanoPVA product) did not have an effect on it. PVP polymers are known for enhancing the aqueous solubility of active pharmaceutical ingredients [25]. Our experiments show that, in the case of papaverine hydrochloride, the addition of PVPK to the original and the micronized substance slightly but significantly increased the equilibrium solubility. Therefore, the solubility enhancement can be attributed to the particle size reduction and the use of PVPK excipient simultaneously.

#### 3.1.2. Dissolution Measurements

**Figure 2:**
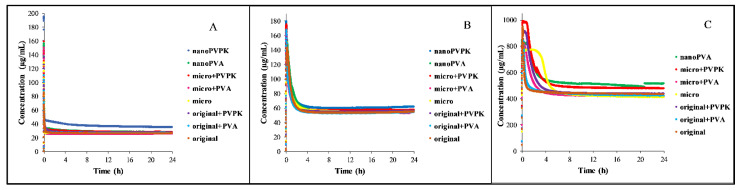
Dissolution profile of papaverine hydrochloride in FaSSIF blank (**A**), FaSSIF full (**B**) and FeSSIF blank (**C**) medium.

The dissolution of the samples in different media is shown in Figure 2. For the better visualization of the supersaturation and the precipitation, the dissolution of papaverine hydrochloride in FaSSIF blank, FaSSIF full and FeSSIF blank media is enlarged and depicted in Figure S3.

**Figure 3:**
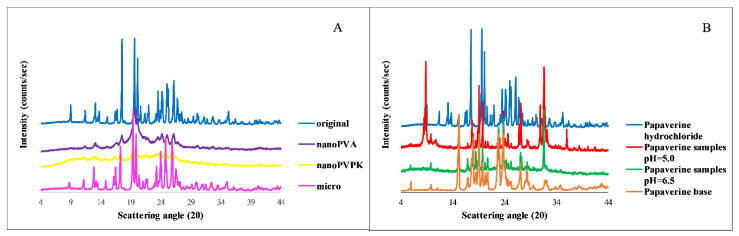
PXRD diffractogram of the original, micronized and nanonized substances (**A**) and furthermore the papaverine samples from the solubility measurements (pH = 6.5 and pH = 5.0) compared to the solid papaverine base and papaverine hydrochloride salt (**B**).

In all media, the samples show a high supersaturation and then precipitation until they reach the equilibrium concentration. Because of the partial amorphization of the nanonized samples (see Figure 3A), the dissolution rate and the supersaturation in these cases are not relevant, but based on the results of the real-time monitoring the time needed to reach the equilibrium can be determined. However, the equilibration time is dependent on the media. In the FaSSIF blank buffer, all the samples are precipitating almost immediately. In the FaSSIF full, which contains SIF powder (sodium taurocholate and lecithin as solubilizing agents), this process takes longer, the time reaching the equilibrium was in between 1 and 4 h. The presence of the SIF powder may be the reason for the longer supersaturation. Because of the ionization state, the solubility of the compound in the FeSSIF blank medium is approximately ten times higher than in the FaSSIF blank buffer. This can explain why the degree of supersaturation is lower than in the FaSSIF buffers and can also be a reason for the longer equilibration. The concentration of the samples in the FeSSIF full medium and the nanoPVPK sample in the FeSSIF blank buffer exceeded the limit of the instrument, therefore no measurements could be performed in these cases.

#### 3.1.3. Particle Size Analysis

Table 3 shows the particle size distribution of the solid phase samples taken from the solubility suspensions at the end of the measurements.

Our results show that the nanonized formulations did not stay in the submicron range at the end of the measurement, as an aggregation can be observed between the particles. In some cases, larger particles were forming from the nanoparticles, then from the original or the micronized initial substances. The particle size of the suspension from the measurement of the original or the micronized compound is consistently smaller than the particle size of the starting compound. In these cases, the presence of the excipients can inhibit the aggregation resulting in a smaller size range. In the pH = 6.5 solutions, where the molecule is approximately 50% in deprotonated state, a smaller particle size is characteristic compared to the pH = 5.0 media.

#### 3.1.4. PXRD

The structure of the original, micronized and nanonized APIs, as well as the solid phase samples from the solubility measurements, were verified by PXRD measurements. The results show that the original and the micronized samples have a crystalline structure. In the case of nanonized compounds an amorphous background can be observed, however, some of the characteristic peaks are still present, therefore these samples are only partially amorphous. Considering the samples from the solubility measurements in pH = 6.5 media, a salt-to-free base form transformation takes place, and the characteristic peaks of the papaverine base appear on the diffractogram belonging to the pH = 6.5 samples. In the case of the pH = 5.0 media, a mixture of free base and hydrochloride salt is present at the end of the measurement. Because the samples with different particle size and excipients were showing the same characteristic peaks, only one diffractogram is shown per media in Figure 3. In the case of nanonized samples, the amorphous background cannot be observed at the end of the measurements. Therefore, the faster dissolution can be attributable to the partial amorphization, but based on the PXRD diffractograms at the end of the measurements, the solution is in equilibrium with a crystalline solid phase. It means that the equilibrium solubility results belong to a crystalline substance, so the probability of the effect of the amorphization on the measured solubility is very low.

### 3.2. Furosemide

#### 3.2.1. Solubility Measurements

**Figure 4:**
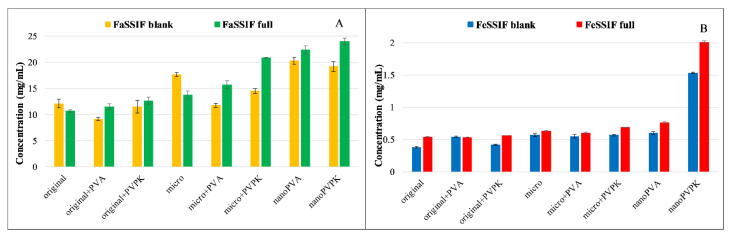
Equilibrium concentration of furosemide solutions in pH = 6.5 (**A**) and pH = 5.0 (**B**) media.

Figure 4 shows the solubility of furosemide in the four different media used for the measurements.

Furosemide is a weak bivalent acid with p*K*_a1_ = 3.53; p*K*_a2_ = 10.15 at 37 °C [26]. At pH = 6.5, where the compound is exclusively in the monoanionic form, its solubility is excellent, more than 10 mg/mL. At pH = 5.0, the ionization of furosemide is somewhat lower, but it is still predominantly ionized (HA^−^: 96.7% H_2_A: 3.3%). This change in the protonation state results in an approximately ten times lower equilibrium concentration. Because of the high solubility, the solubilizing effect of the SIF powder is limited: in the FaSSIF full buffer, it even impairs the solubility of the original and the micronized compound without excipients compared to the blank buffer. In the FeSSIF full, a significant solubility increase can be observed in all samples when it is compared to the blank buffer. Excipients in the physical mixtures of the original and the micronized compounds did not affect, or even slightly decreased the equilibrium concentration in FaSSIF buffers. In the case of nanoPVA and nanoPVPK samples, a solubility enhancement was observable, and there were no significant differences between the two samples. In the FeSSIF buffers, the PVPK excipient resulted in a higher solubility when added to the original and the micronized compound. Nanonization with PVA did not change the solubility, but the nanoPVPK sample had an outstanding equilibrium concentration (1.535 ± 0.0098 mg/mL in FeSSIF blank and 2.007 ± 0.025 mg/mL in FeSSIF full).

#### 3.2.2. Dissolution Measurements

**Figure 5:**
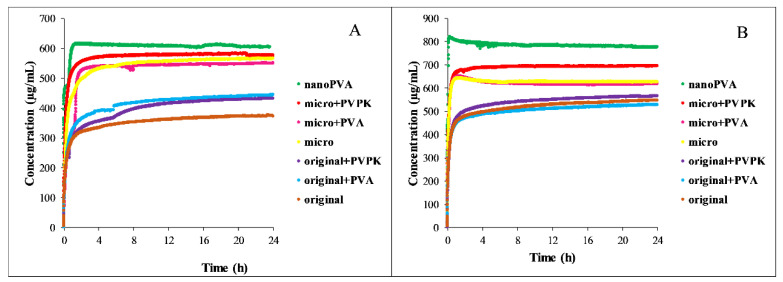
Dissolution profile of furosemide in FeSSIF blank (**A**) and FeSSIF full (**B**) medium.

Measuring the concentration in real time was only possible in the pH = 5.0 buffer. The results are shown in Figure 5, except the nanoPVPK samples, because they were out of the range of the instrument also in these media.

In both media, the nanonized sample showed the fastest dissolution, which can be attributable to the partial amorphous character, but also in this case the results of the real-time monitoring support the selection of the time needed to reach the equilibrium. Supersaturation was also characteristic of its behavior. Nanonization results in a higher solubility than the micronized samples, but in dissolution there was no significant difference between them, both of them show faster dissolution than the original sample. The added excipients did not affect the dissolution, only the equilibrium concentration of the samples.

#### 3.2.3. Particle Size Analysis

The particle size range of the furosemide samples from the end of the solubility measurements is shown in Table 4.

In the pH = 6.5 media, a smaller particle size can be observed. Presumably, in these cases, the negative charge of the anionic form causes a decreased tendency to aggregation. NanoPVPK samples in these media partially stay under the 1 µm limit, but approximately 40% of the particles exceed this size, which refers to aggregation. The results show that PVA cannot prevent aggregation in the case of nanonized samples, therefore a larger particle size is distinctive compared to nanoPVPK samples in the pH = 6.5 media. In the pH = 5.0 buffers, none of the nanonized samples stays in the submicron range. Micronized samples have a smaller particle size before and after the measurements than the original samples. Both the original and the micronized compound with and without excipients show a smaller particle size than the starting compound.

#### 3.2.4. PXRD

**Figure 6:**
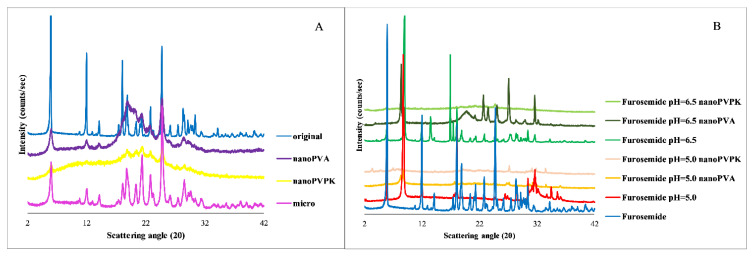
PXRD diffractogram of the original, micronized and nanonized furosemide (**A**) and the samples from the solubility measurements (pH = 6.5 and pH = 5.0) compared to the solid furosemide starting compound (**B**).

Based on the results of the PXRD measurements shown in Figure 6, the original and the micronized compounds have a crystalline structure with the same characteristic peaks. The nanonized samples both have an amorphous background, but to varying degrees: as long as the diffractogram of the nanoPVA sample contains all the characteristic peaks but with a lower intensity, the diffractogram of the nanoPVPK sample only contains characteristic peaks in the 15–25 2θ range. The isolated solid phase from the solubility measurements in the pH = 6.5 buffers shows that the original and the micronized samples with and without excipients were going through a free acid-to-salt form transformation: the appearing peak at 8.8 2θ while the one at 5.9 2θ is disappearing is characteristic of the furosemide sodium salt [27]. In the pH = 5.0 buffers, the same salt transformation can be observed based on the PXRD diffractograms. The diffractogram of the original and the micronized substances with and without excipients showed the same characteristic peaks; only one diffractogram per pH is shown next to the solid furosemide and the nanonized compounds. In the case of the nanonized samples, the amorphous character is also observable at the end of the measurements in all media; however, the characteristic peaks are appearing on the diffractogram but with low intensity. Therefore, the higher equilibrium solubility results can be attributable to the combined effect of the particle size reduction and the amorphous character [28,29].

### 3.3. Niflumic Acid

#### 3.3.1. Solubility Measurements

**Figure 7:**
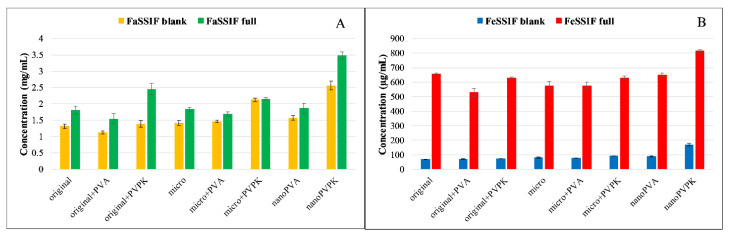
Solubility of niflumic acid in FaSSIF blank/full (**A**) and FeSSIF blank/full (**B**) buffers.

The solubility results of niflumic acid are shown in Figure 7.

Niflumic acid is an ampholyte with an acidic carboxyl group and with an aminopyridinyl function, which is a weak base (p*K*_a1_ = 2.26; p*K*_a2_ = 4.44). Knowing the protonation microconstants, the relative concentration of the particles can be calculated for each of the pH values [30]. At pH = 5.0, niflumic acid is present in 78.2% anionic, 20.6% zwitterionic and 1.2% neutral form. This results in approximately ten times lower solubility than in the pH = 6.5 buffers, where niflumic acid is predominantly anionic (99.1%). In the FeSSIF blank buffer, the presence of the excipient as a physical mixture did not affect the solubility of the original and the micronized compound. Micronization did not change the equilibrium concentration, and also out of the nanonized samples only the nanoPVPK showed a significantly increased solubility. Adding the SIF powder to the FeSSIF blank buffer resulted in a drastically increased solubility, the solubilizing effect was much more pronounced than in the FaSSIF blank/full buffers. In these media, the solubilizing effect of the PVPK excipient could be observed: it enhanced the solubility of the original and the micronized compound. In contrast, PVA as excipient did not change significantly or slightly decreased the solubility of the niflumic acid in both original, micronized and nanonized forms. The nanoPVPK sample still had the highest solubility of all samples, thanks to the effect of the nanonization and the excipient.

#### 3.3.2. Dissolution Measurements

**Figure 8:**
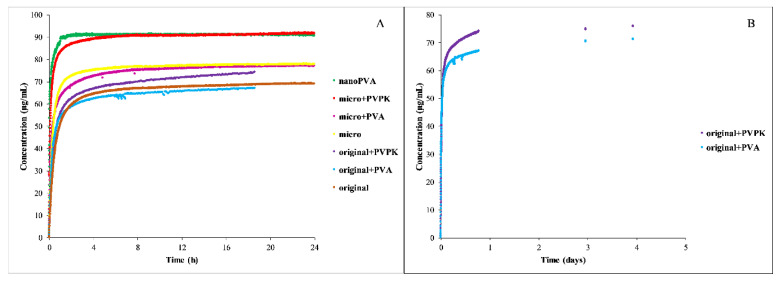
Dissolution profile of all niflumic acid samples (**A**) and the physical mixtures of the original compound with excipients (**B**) in FeSSIF blank buffers.

Because of the limit of the instrument, the dissolution of the niflumic acid could be monitored in real-time in the FeSSIF blank buffer—except the nanoPVPK sample, which also had a solubility over the limit in this media. The results of the dissolution measurements are shown in Figure 8.

**Figure 9:**
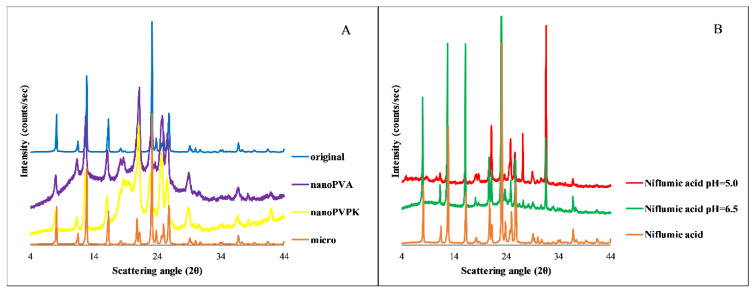
PXRD diffractogram of the original, micronized and nanonized niflumic acid (**A**) and the samples from the solubility measurements (pH = 6.5 and pH = 5.0) compared to the solid niflumic acid starting compound (**B**).

The results show that the particle size reduction resulted in a faster dissolution, the excipients only affected the equilibrium solubility of the micronized and nanonized compounds. In the case of the nanoPVA sample, the fast dissolution can be the result of the partial amorphous character. The B panel of Figure 9 shows the importance of the real-time monitoring, because the excipients increased the time needed to reach the equilibrium in the case of the original compound: 24 h were not enough, it lasted for days.

#### 3.3.3. Particle Size Analysis

The results of the particle size analysis of the niflumic acid samples after the measurements are shown in Table 5.

Similar to the behavior of the furosemide, in the pH = 6.5 buffer, where the molecules are in an ionized state, a smaller particle size can be observed because of the inhibited aggregation. This phenomenon also can be observed when the particle size of the samples without excipients is compared to the samples with excipients: the presence of both PVA and PVPK results in a smaller particle size range. The nanonized samples did not stay in the submicron range, but in the presence of PVPK a smaller particle size can be observed, so it can prevent aggregation more efficiently. The particle size of the suspension from the measurement of the original and the micronized compound with and without excipients is always smaller than the particle size of the starting compound.

#### 3.3.4. PXRD

The solid phase analysis after the solubility measurements was performed by PXRD; the results are shown in Figure 9.

The A panel of Figure 9 shows that the micronized sample has the same characteristic peaks as the original niflumic acid. In the case of nanonized compounds, an amorphous background can be observed, but the characteristic peaks are present, which prove the crystalline structure. The diffractogram of the niflumic acid from both the pH = 5.0 and pH = 6.5 measurements compared to the original niflumic acid shows the same characteristic peaks, which proves that the compound does not transform during the solubility measurements. In the case of nanonized samples, the amorphous background is negligible at the end of the solubility measurements, the characteristic peaks of the crystalline substance are appearing on the diffractogram. Therefore, the faster dissolution can be attributable to the partial amorphization, but based on the PXRD diffractograms at the end of the measurements, the solution is in equilibrium with a crystalline solid phase, such as in the case of papaverine hydrochloride.

## 4. Conclusions

This work provides a comprehensive study of the effect of the particle size reduction, pH, excipients and solubilizing agents on the solubility and the dissolution of drugs with different acid-base characters. It was proved that micronization does not change the equilibrium solubility of a drug, but it results in a faster dissolution. In contrast, nanonization can improve the equilibrium solubility of a drug (out of the investigated compounds, papaverine hydrochloride and niflumic acid), but it depends on the excipient used for nanonization. Out of the two investigated polymer excipients, PVA as a physical mixture next to the original or the micronized substance either did not affect or eventually decreased the solubility of the drugs. This negative effect can be the explanation for the different behavior of the two nanonized samples: nanonization alone can improve the solubility, but the PVA compensates for this effect. In contrast, PVPK as a physical mixture improved the solubility in most of the cases. The reason for this difference between the two polymers is the presence of the different functional groups on the monomers capable of interactions with API. PVP K25 is able to form both polar (H-bonds on O and N atoms of pyrrolidone function) and apolar hydrophobic interactions (van der Waals forces with the apolar site of the ring), while for PVA only H-bond forming is probable. Therefore, the exceptionally high solubility of the nanoPVPK samples of niflumic acid and papaverine hydrochloride resulted from the impact of the nanonization and the solubilizing effect of the polymer excipient. The excipients also have a significant role regarding to the particle size at the end of the solubility measurements: they can prevent aggregation in the case of micronized samples. However, this effect is not strong enough to hold the nanonized samples in the submicron state. PVPK is also more effective in this perspective. The effect of the pH on the solubility depends on the p*K*_a_ of the molecules. In biorelevant media, the solubilizing effect of the bile salts and lecithin can be observed in the case of all investigated media and APIs. It can be concluded that the use of biorelevant media is an important influencing parameter regarding the solubility or dissolution. However, the effect of ionization on the solubility can be more pronounced, in these cases the effect of the solubilizing agents of biorelevant media is less influencing, so using a FaSSIF or FeSSIF full buffer is more important, when the substances are in a neutral state at pH = 6.5 or pH = 5.0. This work claims that nanonization can be an effective method to improve the solubility, but the selection of the excipients to prepare these samples can greatly influence the performance of the formulation. During the development of a drug, using biorelevant media to investigate the solubility of an API is strongly recommended, because the pH alone cannot model the conditions prevailing in the gastrointestinal tract—in extreme cases, the solubilizing effect of the bile salts and lecithin makes an order of magnitude difference in the solubility of a drug.

## Figures and Tables

**Table 1:** Composition of the investigated formulations.

Composition of the Formulation	Nomination
commercially available substance without excipient	original
physical mixture of the commercially available substance and PVA in 1:1 mass ratio	original + PVA
physical mixture of the commercially available substance and PVPK-25 in 1:1 mass ratio	original + PVPK
micronized sample without excipient	micro
physical mixture of the micronized substance and PVA in 1:1 mass ratio	micro + PVA
physical mixture of the micronized substance and PVPK-25 in 1:1 mass ratio	micro + PVPK
nanonized sample with PVA excipient	nanoPVA
nanonized sample with PVPK-25 excipient	nanoPVPK

**Table 2:** Particle size distribution of the original and the micronized compounds.

		d = 0.1 (µm)	d = 0.5 (µm)	d = 0.9 (µm)
PAP	original	153.97	488.62	887.93
	micro	101.71	263.70	596.47
FUR	original	7.36	62.46	294.13
	micro	14.97	58.94	148.10
NIF	original	71.45	183.25	579.83
	micro	38.91	110.46	224.97

**Table 3:** Particle size distribution of the solid phase samples from the solubility measurements of papaverine hydrochloride.

		pH = 6.5			pH = 5.0		
		d = 0.1 (µm)	d = 0.5 (µm)	d = 0.9 (µm)	d = 0.1 (µm)	d = 0.5 (µm)	d = 0.9 (µm)
PAP	original	7.96	33.39	98.40	4.60	49.42	189.96
	original + PVA	6.75	23.57	85.95	5.40	34.02	126.18
	original + PVPK	10.92	28.29	57.42	21.55	53.25	98.98
	micro	13.22	38.17	76.99	8.22	66.08	129.94
	micro + PVA	7.14	21.37	46.42	8.35	40.53	91.06
	mikro + PVPK	10.43	27.87	57.36	18.90	47.35	88.34
	nanoPVA	12.56	55.80	143.53	23.90	80.71	160.25
	nanoPVPK	7.77	37.98	113.67	29.73	62.00	120.43

**Table 4:** Particle size distribution of the solid phase samples from the solubility measurements of furosemide.

		pH = 6.5			pH = 5.0		
		d = 0.1 (µm)	d = 0.5 (µm)	d = 0.9 (µm)	d = 0.1 (µm)	d = 0.5 (µm)	d = 0.9 (µm)
FUR	original	4.72	14.63	35.04	16.24	51.23	110.56
	original + PVA	8.46	35.98	97.24	8.40	31.78	83.27
	original + PVPK	6.04	34.83	94.75	9.53	35.88	88.28
	micro	3.05	10.16	25.98	1.90	7.36	24.69
	micro + PVA	1.91	5.10	11.12	3.41	14.05	91.50
	micro + PVPK	1.51	1.75	9.51	2.29	16.92	50.15
	nanoPVA	4.56	22.61	66.78	7.18	32.44	89.24
	nanoPVPK	0.34	0.78	2.16	1.45	75.81	305.43

**Table 5:** Particle size distribution of the solid phase samples from the solubility measurements of niflumic acid.

		pH = 6.5			pH = 5.0		
		d = 0.1 (µm)	d = 0.5 (µm)	d = 0.9 (µm)	d = 0.1 (µm)	d = 0.5 (µm)	d = 0.9 (µm)
NIF	original	19.07	58.55	128.24	56.21	151.25	488.95
	original + PVA	8.29	38.74	112.33	9.97	47.10	126.18
	original + PVPK	11.39	42.73	95.69	13.60	66.44	164.08
	micro	8.59	32.98	96.05	16.57	71.37	164.93
	micro + PVA	4.71	18.60	94.05	7.61	52.11	147.07
	micro + PVPK	1.94	3.53	11.09	2.73	15.18	64.21
	nanoPVA	10.52	47.76	121.88	10.71	50.03	137.71
	nanoPVPK	1.98	3.34	9.48	1.33	5.26	17.26

## Data Availability

All data can be provided by the authors upon request. No publicly accessible archive storage is available.

## Supplementary Materials

The following supporting information can be downloaded at: https://www.mdpi.com/article/10.3390/pharmaceutics15010278/s1, Figure S1: Calibration curve of furosemide for the calculation of the specific absorbance in FaSSIF blank media; Figure S2: SEM images of papaverine hydrochloride nanonized with PVPK (A) and PVA (B); furosemide nanonized with PVPK (C) and PVA (D); niflumic acid nanonized with PVPK (E) and PVA (F); Figure S3: Dissolution profile of papaverine hydrochloride in FaSSIF blank (A), FaSSIF full (B) and FeSSIF blank (C) medium, the time on the x axis is adjusted to the time of precipitation; Table S1. Mean particle size of the nanonized compounds.

## References

[1] Amidon G.L., Lennernäs H., Shah V.P., Crison J.R. (1995). A Theoretical Basis for a Biopharmaceutic Drug Classification: The Correlation of in Vitro Drug Product Dissolution and in vivo Bioavailability. Pharm. Res..

[2] Bergström C.A.S., Box K., Holm R., Matthews W., McAllister M., Müllertz A., Rades T., Schäfer K.J., Teleki A. (2019). Biorelevant intrinsic dissolution profiling in early drug development: Fundamental, methodological, and industrial aspects. Eur. J. Pharm. Biopharm..

[3] Savjani K.T., Gajjar A.K., Savjani J.K. (2012). Drug Solubility: Importance and Enhancement Techniques. ISRN Pharm..

[4] Jain S., Patel N., Lin S. (2015). Solubility and dissolution enhancement strategies: Current understanding and recent trends. Drug Dev. Ind. Pharm..

[5] Ainurofiq A., Putro D.S., Ramadhani D.A., Putra G.M., Santo L.D.D. (2021). A review on solubility enhancement methods for poorly water-soluble drugs. J. Rep. Pharm. Sci..

[6] Khadka P., Ro J., Kim H., Kim I., Kim J.T., Kim H., Cho J.M., Yun G., Lee J. (2014). Pharmaceutical particle technologies: An approach to improve drug solubility, dissolution and bioavailability. Asian J. Pharm. Sci..

[7] Szunyogh T., Ambrus R., Szabó-Révész P. (2013). Nanonization of Niflumic Acid by Co-Grinding. Adv. Nanopart..

[8] LeLeux J., Williams R.O. (2014). Recent advancements in mechanical reduction methods: Particulate systems. Drug Dev. Ind. Pharm..

[9] Wais U., Jackson A.W., He T., Zhang H. (2015). Nanoformulation and encapsulation approaches for poorly water-soluble drug nanoparticles. Nanoscale.

[10] Prajapati H., Serajuddin A.M. (2022). Development of Fully Redispersible Dried Nanocrystals by Using Sucrose Laurate as Stabilizer for Increasing Surface Area and Dissolution Rate of Poorly Water-Soluble Drugs. J. Pharm. Sci..

[11] Csicsák D., Borbás E., Kádár S., Tőzsér P., Bagi P., Pataki H., Sinkó B., Takács-Novák K., Völgyi G. (2021). Towards more accurate solubility measurements with real time monitoring: A carvedilol case study. New J. Chem..

[12] Avdeef A., Fuguet E., Llinàs A., Ràfols C., Bosch E., Völgyi G., Verbić T., Boldyreva E., Takács-Novák K. (2016). Equilibrium solubility measurement of ionizable drugs—Consensus recommendations for improving data quality. ADMET DMPK.

[13] Kaptay G. (2012). On the size and shape dependence of the solubility of nano-particles in solutions. Int. J. Pharm..

[14] Clarysse S., Brouwers J., Tack J., Annaert P., Augustijns P. (2011). Intestinal drug solubility estimation based on simulated intestinal fluids: Comparison with solubility in human intestinal fluids. Eur. J. Pharm. Sci..

[15] Jantratid E., Janssen N., Reppas C., Dressman J.B. (2008). Dissolution Media Simulating Conditions in the Proximal Human Gastrointestinal Tract: An Update. Pharm. Res..

[16] Fagerberg J.H., Tsinman O., Sun N., Tsinman K., Avdeef A., Bergström C.A.S. (2010). Dissolution Rate and Apparent Solubility of Poorly Soluble Drugs in Biorelevant Dissolution Media. Mol. Pharm..

[17] Baka E., Comer J.E.A., Takács-Novák K. (2008). Study of equilibrium solubility measurement by saturation shake-flask method using hydrochlorothiazide as model compound. J. Pharm. Biomed..

[18] He Y., Ho C., Yang D., Chen J., Orton E. (2017). Measurement and Accurate Interpretation of the Solubility of Pharmaceutical Salts. J. Pharm. Sci..

[19] Saifee M., Inamda N., Dhamecha D.L., Rathi A.A. (2009). Drug polymorphism: A review. Int. J. Health Res..

[20] Takács-Novák K., Szőke V., Völgyi G., Horváth P., Ambrus R., Szabó-Révész P. (2013). Biorelevant solubility of poorly soluble drugs: Rivaroxaban, furosemide, papaverine and niflumic acid. J. Pharm. Biomed..

[21] Bijlani V., Yuonayel D., Katpally S., Chukwumezie B.N., Adeyeye M.C. (2007). Monitoring ibuprofen release from multiparticulates: In situ fiber-optic technique versus the HPLC method: A technical note. AAPS PharmSciTech.

[22] Bynum K., Roinestad K., Kassis A., Pocreva J., Gehrlein L., Cheng F., Palermo P. (2001). Analytical Performance of a Fiber Optic Probe Dissolution System. Dissolution Technol..

[23] Gray V.A. (2003). Dissolution Testing Using Fiber Optics—A Regulatory Perspective. Dissolution Technol..

[24] Völgyi G., Csicsák D., Takács-Novák K. (2018). Right filter-selection for phase separation in equilibrium solubility measurement. Eur. J. Pharm. Sci..

[25] Kurakula M., Rao G.S.N.K. (2020). Pharmaceutical assessment of polyvinylpyrrolidone (PVP): As excipient from conventional to controlled delivery systems with a spotlight on COVID-19 inhibition. J. Drug Deliv. Sci. Technol..

[26] Sun N., Avdeef A. (2011). Biorelevant pKa (37 °C) predicted from the 2D structure of the molecule and its pKa at 25 °C. J. Pharm. Biomed..

[27] Khandavilli U.B.R., Gangavaram S., Goud N.R., Cherukuvada S., Raghavender S., Nangia A., Manjunatha S.G., Nambiar S., Pal S. (2014). High solubility crystalline hydrates of Na and K furosemide salts. CrystEngComm.

[28] Kádár S., Csicsák D., Tőzsér P., Farkas A., Pálla T., Mirzahosseini A., Tóth B., Tóth G., Fiser B., Horváth P. (2022). Understanding the pH Dependence of Supersaturation State—A Case Study of Telmisartan. Pharmaceutics.

[29] Hancock B.C., Parks M. (2000). What is the True Solubility Advantage for Amorphous Pharmaceuticals?. Pharm. Res..

[30] Takács-Novák K., Avdeef A., Box K.J., Podányi B., Szász G. (1995). Determination of protonation macro- and microconstants and octanol/water partition coefficient of the antiinflammatory drug niflumic acid. J. Pharm. Biomed..

