# Supplementary material for: The Effect of the Particle Size Reduction on the Biorelevant Solubility and Dissolution of Poorly Soluble Drugs with Different Acid-Base Character
Source: Pharmaceutics. 2023 Jan 13;15(1):278. doi: 10.3390/pharmaceutics15010278 (PMC9865396; doi:10.3390/pharmaceutics15010278)
Supplement: Supplementary file 1 [file pharmaceutics-15-00278-s001.zip › pharmaceutics-1990667-supplementary.pdf]

pharmaceutics

Supplementary Materials: The Effect of the Particle Size Reduction on the Biorelevant Solubility and Dissolution of Poorly Soluble Drugs with Different Acid-Base Character

Dóra Csicsák [1], Rita Szolláth [1], Szabina Kádár [2], Rita Ambrus [3], Csilla Bartos [3], Emese Balogh [4], István Antal [4], István Köteles [1], Petra Tőzsér [2], Vivien Bárdos [1], Péter Horváth [1], Enikő Borbás [2], Krisztina Takács-Novák [1], Bálint Sinkó [5] and Gergely Völgyi [1,*]

[1] Department of Pharmaceutical Chemistry, Semmelweis University, 9 Hőgyes Endre Street, 1092 Budapest, Hungary
[2] Department of Organic Chemistry and Technology, Faculty of Chemical Technology and Biotechnology, Budapest University of Technology and Economics, 3 Műegyetem Rkp., 1111 Budapest, Hungary
[3] Institute of Pharmaceutical Technology and Regulatory Affairs, University of Szeged, 6 Eötvös Street, 6720 Szeged, Hungary
[4] Department of Pharmaceutics, Semmelweis University, 7 Hőgyes Endre Street, 1092 Budapest, Hungary
[5] Pion Inc., 10 Cook Street, Billerica, MA 01821, USA
* Correspondence: volgyi.gergely@pharma.semmelweis-univ.hu

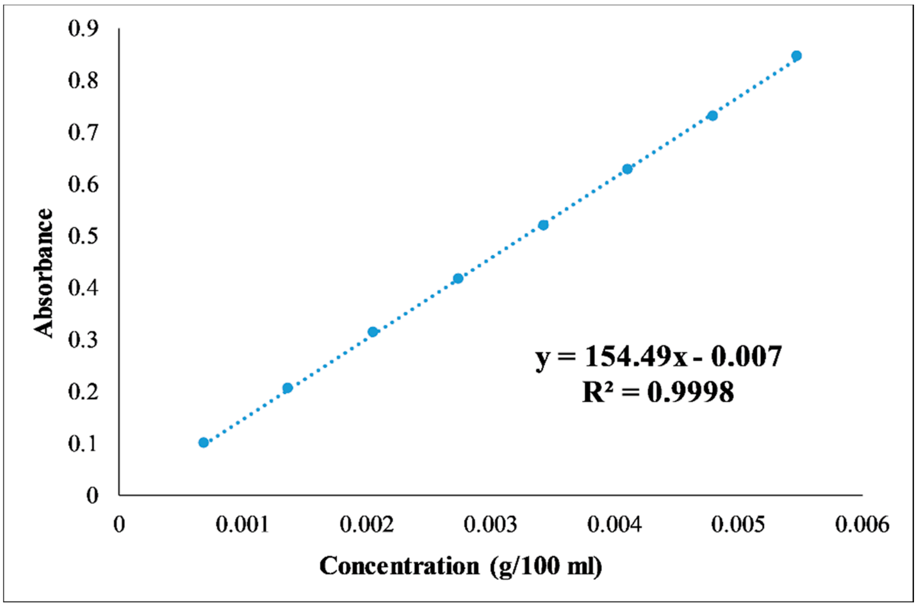

Figure S1: Calibration curve of furosemide for the calculation of the specific absorbance in FaSSIF blank media

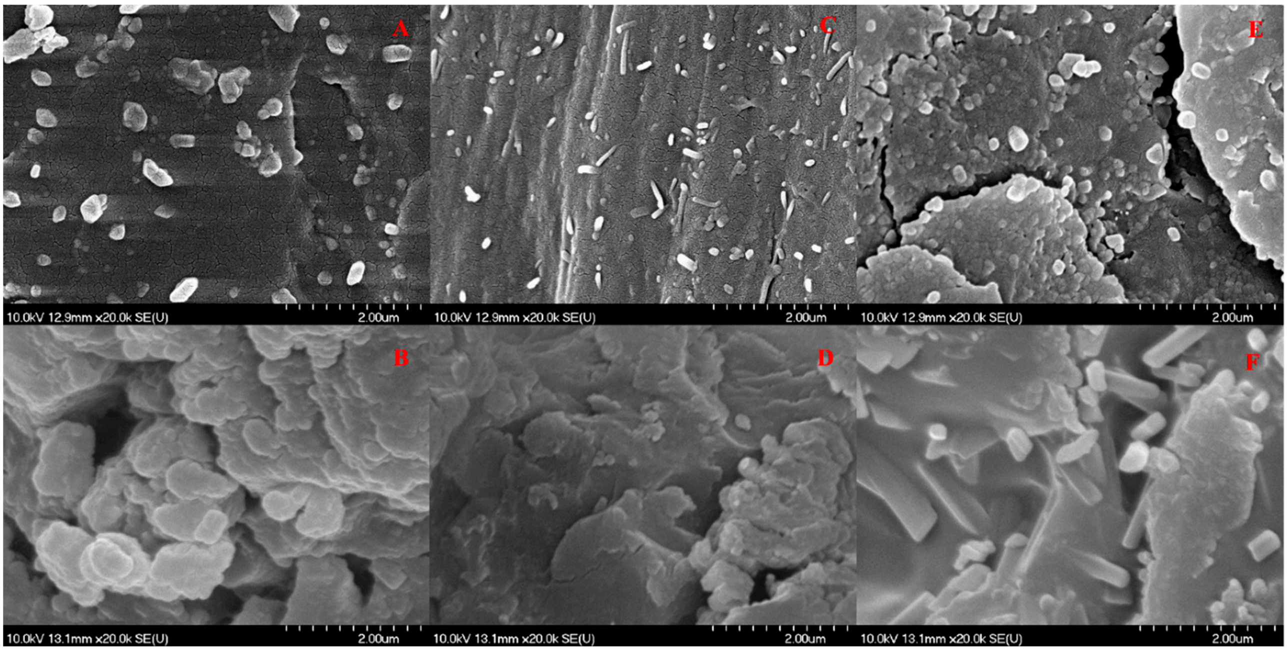

Figure S2. SEM images of papaverine hydrochloride nanonized with PVPK (A) and PVA (B); furosemide nanonized with PVPK (C) and PVA (D); niflumic acid nanonized with PVPK (E) and PVA (F)

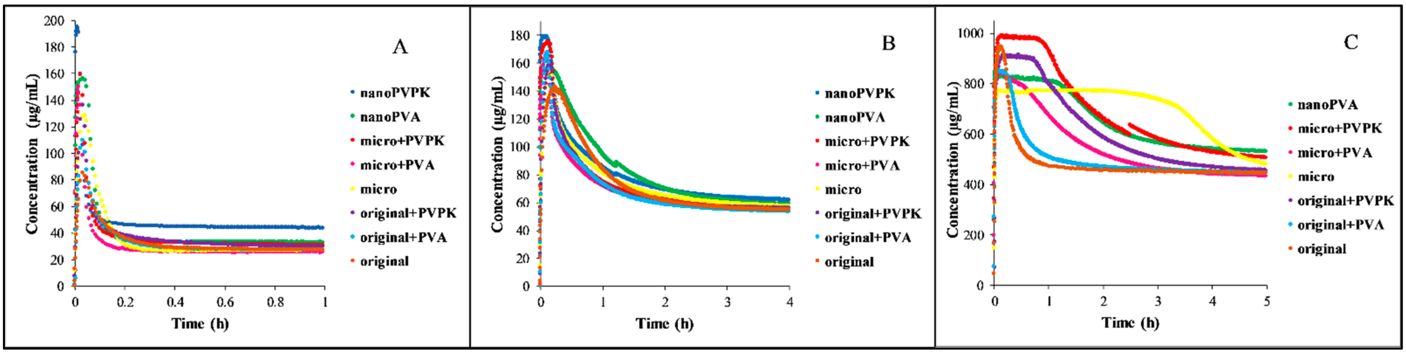

Figure S3. Dissolution profile of papaverine hydrochloride in FaSSIF blank (A), FaSSIF full (B) and FeSSIF blank (C) medium, the time on the x axis is adjusted to the time of precipitation

Table S1. Mean particle size of the nanonized compounds.

	A	B	C	D	E	F
Mean (nm)	288.3	225.4	305.8	229.8	212.2	743.3
SD±	116.1	96.9	104.7	67.3	47.2	720.9
